# Pasteurella multocida toxin – lessons learned from a mitogenic toxin

**DOI:** 10.3389/fimmu.2022.1058905

**Published:** 2022-12-16

**Authors:** Katharina F. Kubatzky

**Affiliations:** Department of Infectious Diseases, Medical Microbiology and Hygiene, Heidelberg University, Heidelberg, Germany

**Keywords:** Pasteurella multocida, exotoxin, immune system, G protein, osteoclast, osteoblast, signaling

## Abstract

The gram-negative, zoonotic bacterium *Pasteurella multocida* was discovered in 1880 and found to be the causative pathogen of fowl cholera. *Pasteurella*-related diseases can be found in domestic and wild life animals such as buffalo, sheep, goat, deer and antelope, cats, dogs and tigers and cause hemorrhagic septicemia in cattle, rhinitis or pneumonia in rabbits or fowl cholera in poultry and birds. *Pasteurella multocida* does not play a major role in the immune-competent human host, but can be found after animal bites or in people with close contact to animals. Toxigenic strains are most commonly found in pigs and express a phage-encoded 146 kDa protein, the *Pasteurella multocida* toxin (PMT). Toxin-expressing strains cause atrophic rhinitis where nasal turbinate bones are destroyed through the inhibition of bone building osteoblasts and the activation of bone resorbing osteoclasts. After its uptake through receptor-mediated endocytosis, PMT specifically targets the alpha subunit of several heterotrimeric G proteins and constitutively activates them through deamidation of a glutamine residue to glutamate in the alpha subunit. This results in cytoskeletal rearrangement, proliferation, differentiation and survival of cells. Because of the toxin’s mitogenic effects, it was suggested that it might have carcinogenic properties, however, no link between *Pasteurella* infections and cell transformation could be established, neither in tissue culture models nor through epidemiological data. In the recent years it was shown that the toxin not only affects bone, but also the heart as well as basically all cells of innate and adaptive immunity. During the last decade the focus of research shifted from signal transduction processes to understanding how the bacteria might benefit from a bone-destroying toxin. The primary function of PMT seems to be the modulation of immune cell activation which at the same time creates an environment permissive for osteoclast formation. While the disease is restricted to pigs, the implications of the findings from PMT research can be used to explore human diseases and have a high translational potential. In this review our current knowledge will be summarized and it will be discussed what can be learned from using PMT as a tool to understand human pathologies.

## 1 Introduction

### 1.1 Diseases caused by *Pasteurella multocida* infections

*Pasteurella multocida* is a zoonotic bacterium (1). In many animals, members of the *Pasteurellae* genus (including *P. multocida*, *P. canis*, *P. stomatis*, *P. dagmatis*, *P. gallinarum*, *P. avium*, *P. volantium*, *P. langaa* and *P. anatis*) are commensals of the upper respiratory tract that do not normally cause disease (2). *Pasteurella multocida* is an opportunistic family member that can turn pathogenic when the host’s immune competence changes or when the bacteria are able to invade the tissue due to scratches, bites or other lesions. Affected species include but are not limited to cats, dogs, cattle, pigs, goat, deer, poultry and rabbits (3). In humans, *P. multocida* infections are rare and often linked to household pets or the work environment (farmers). Potential diseases include septic bite wounds and inflammation at the site of injury (4) or pneumonia if the bacteria were inhaled (5).

*P. multocida* strains can be grouped into different serogroups based on the composition of the capsule (A, B, D, E and F) that can be further classified into 16 serotypes based on the expressed LPS antigen (6, 7). Due to the protection of capsule possessing strains from innate immune cells, these strains are more virulent than non-capsule strains and evade host immune recognition and show decreased phagocytosis (8). While B and E strains cause hemorrhagic septicemia in cattle and buffalo, avian cholera of poultry is caused by A and F strains. A and D strains are connected with snuffles in rabbits, and pneumonia in cattle, sheep and pigs. Atrophic rhinitis is limited to pigs and to a lesser extent to rabbits and involves toxigenic strains that express *P. multocida* toxin (9, 10). *ToxA*, the gene for PMT, has been reported to originate from a lysogenic phage, as the GC content of that sequence is lower than that of the surrounding DNA (11). Comparative genomics of the toxA gene showed that expression of the gene is rather limited and seems to be evolutionary not favorable (12). It is still unclear if and how the prophage is activated to express ToxA. Also, it remains to be investigated if produced virus particles are able to infect other bacteria. Infection of pigs with toxigenic *P. multocida* causes porcine atrophic rhinitis, a disease characterized by a defect in bone formation causing a twisted snout. In a rat model, subcutaneous injection of PMT caused severe inflammation, weight loss and liver necrosis (13). More recently, the systemic action of PMT in mice was investigated after intraperitoneal injection (14). PMT intoxication was also found in spleen, lungs, thymus, gonads, heart, bone and liver which would reflect the organs and tissues that PMT can potentially target in a host if the infection does not stay local. The effects of systemic *P. multocida* infections with toxigenic strains has been investigated in various pig models and showed an increased proliferation of the bladder epithelium and the ureter tissue (15).

*Pasteurella* infections in general can be treated with antibiotics (16) and protection against toxigenic strains is achieved by an early vaccination of pigs (17). Despite effective vaccination programs, the disease can spread rapidly under modern farming conditions and causes a massive economic loss due to growth retardation. As for other diseases that are caused predominantly by the effect of an exotoxin, PMT should constitute a good antigen for efficient antibody production that would allow toxin neutralization before cell entry (18). However, early investigations showed that the full-length toxin did not lead to immunity and that either the toxoid or a catalytically inactive form of the toxin needed to be used (19). Whether this is due to poor antigenicity or the immune escape effects of the toxin on immune cells that defer a solid T and B cell response is unclear. However, the fact that the single point mutation C1165S that renders the toxin catalytically inactive increases the ability to produce antibodies rather argues for an immune modulating effect of the toxin than poor antigenicity (20). Interestingly, whole cell bacterins are able to reduce the level of colonization by *Pasteurella* bacteria, but only toxoid vaccines are able to protect the animals efficiently from atrophic rhinitis. Because it is difficult and expensive to produce large amounts of the toxin, the search for an optimized and cheaper vaccine is still ongoing (21–23).

### 1.2 *Pasteurella multocida* toxin

Pasteurella multocida toxin (PMT) is a single chain protein with a molecular weight of 146 kDa. The protein can be divided into a receptor binding domain located at the N-terminus (B part) and the C-terminal domain that harbors the catalytic activity (A part). PMT is able to translocate into most cells and therefore the uptake mechanism likely involves a receptor that is ubiquitously expressed. It was suggested that membrane phospholipids play a role in the process (24), but more recently, a CRISPR screen highlighted LDL receptor related protein (LRP1) as an important protein for the uptake of PMT (25). The crystal structure showed that the C-terminal domain consists of three different domains. The domain C1 is attached to the plasma membrane, where it binds to lipids such as phosphatidylserine, phosphatidylglycerol, phosphatidic acid, phosphatidylinositol and phosphatidylinositol 4,5-bisphosphate (PIP_2_) (26). Domain C3 harbors the catalytic activity in form of a thiol protease-like catalytic triad, but the role of the C2 domain has not been identified so far (27). If an exotoxin has a target at the plasma membrane (Rho GTPases or heterotrimeric G proteins), there is a need to provide a mechanism that allows the transport of the toxin after its release from the lysoendosome to the membrane. This is achieved by a structural fold called 4-helix bundle membrane (4HBM) that allows interaction with the phospholipids of the membrane bilayer (28, 29).

**Figure 1:**
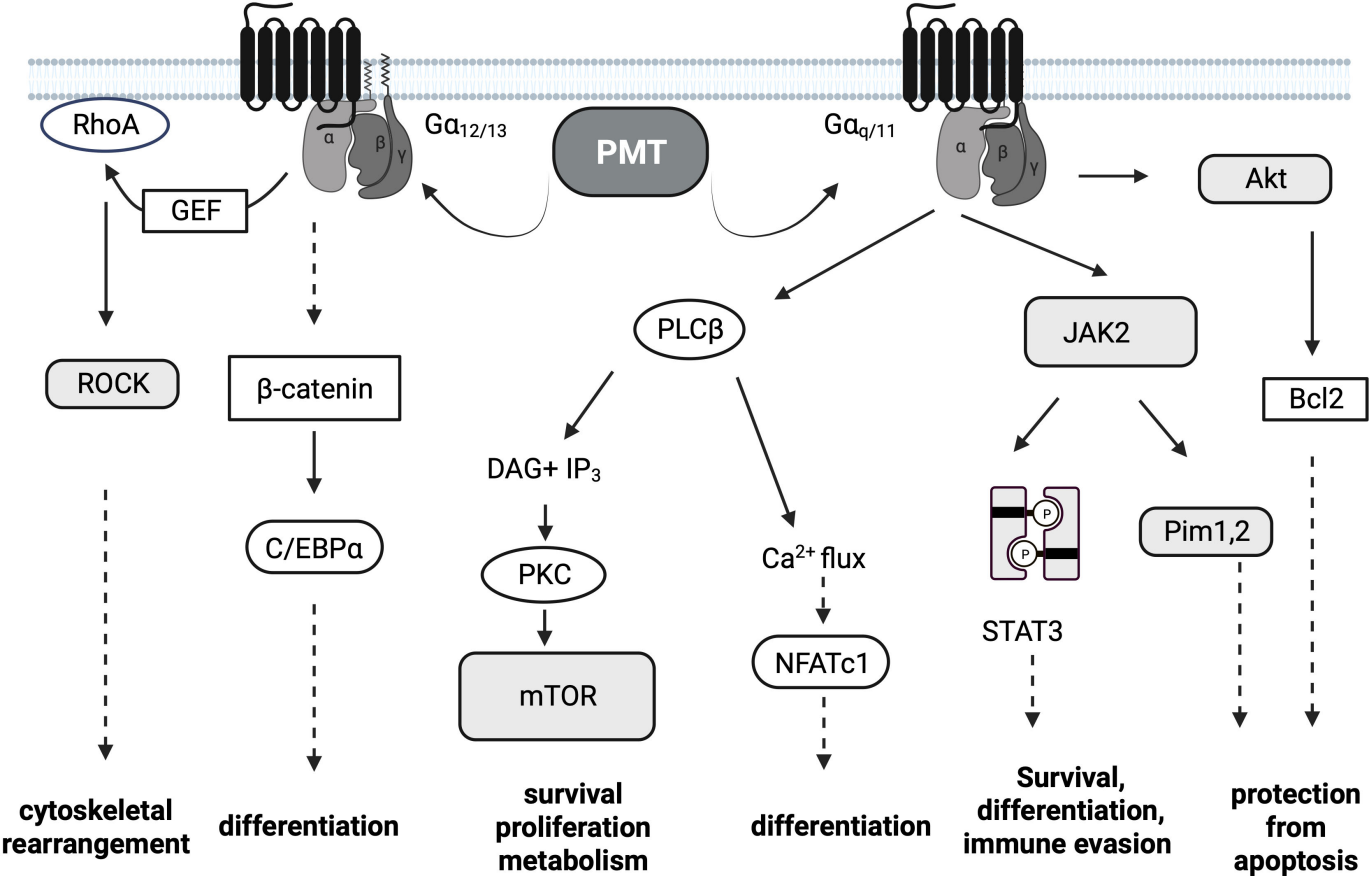
PMT Signaling. *Pasteurella multocida* toxin (PMT)-dependent signaling pathways. PMT modifies the Gα subunits of the heterotrimeric G proteins Gαi, Gαq/11 and Gα12/13 through deamidation. The major subsequent signaling pathways downstream of Gαq/11 and Gα12/13 are depicted. Transcription factors are depicted in white oval boxes, kinases in shaded boxes. Dotted arrows are used if the activation of the depicted molecules is not directly connected.

**Figure 2:**
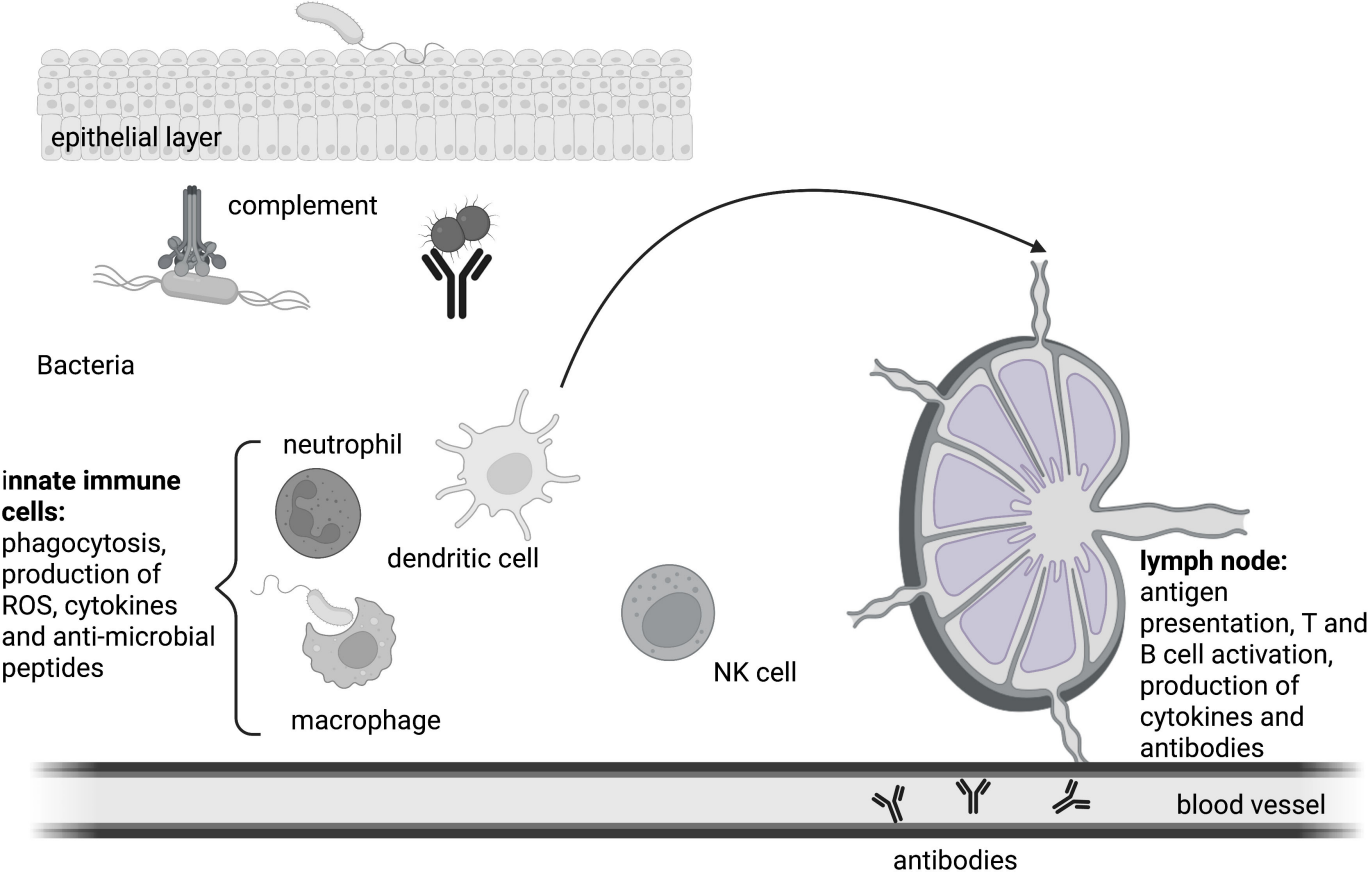
The immune response. Innate immune cells coordinate the early phase of the immune response. Mechanical and biological barriers shield the body from the intrusion of pathogens. Humoral as well as cellular mechanisms defend the integrity of the host upon a bacterial infection. Antigens are presented by professional antigen presenting cells to T cells in the lymph node, ultimately resulting in the formation of germinal centers and the generation of class-switched antibodies by B cells. Antibodies also take part in innate humoral responses like phagocytosis.

Once active PMT is released from the late endosome into the cytosol (30), PMT targets the alpha-subunits of specific heterotrimeric G proteins at the plasma membrane and modifies a glutamine residue to glutamic acid *via* its deamidase activity (31, 32). In contrast to other bacterial toxins like cholera toxin or pertussis toxin that target only one specific G protein, the substrate specificity of PMT is rather broad. Mass spectrometry approaches determined that PMT deamidates Gα_q_/Gα_11_ and Gα_12_/Gα_13_ as well as the Gα_i_ members Gα_i1-3_, while Gαs is not a substrate (31, 33). The resulting modification of the alpha-subunit causes a conformational shift in the protein which renders the G protein constitutively activated. Classical G protein signaling pathways are triggered and result in the stimulation of survival pathways, metabolic activity, proliferation and differentiation (**Figure 1**). Gα_12_/Gα_13_ activation is mainly responsible for the observed cytoskeletal changes through increased Rho GTPase activity, while Gα_i_ deamidation inhibits adenylate cyclase-mediated cAMP production and PKA activation (34). The activation of Gα_q_ seems to be central for many of the observed mitogenic phenomena. Constitutive activation of Gα_q_ by PMT triggers the activation of the transcription factor NFATc1 through calcium release downstream of PLCβ activation (35), activation of JAK-STAT signaling (36) as well as PKC-mediated activation of the serine/threonine kinase mTOR (**Figure 2**) (37). Interestingly, also the release of the Gβγ subunit is part of PMT-induced downstream signaling events and phosphoinositide 3-kinase (PI3K) γ activation results in the production of phosphatidylinositol-3,4,5-trisphosphate (PIP3) (38).

PMT has been suggested by several researchers to act as a potential carcinogen (39, 40). Viral carcinogens can be recognized as the driving cause of cancer due to the occurrence of a constitutively active viral form of a cellular protein, as for example observed for Rous Sarcoma Virus (41). This is more difficult for bacteria, where there is only limited evidence to directly link the development of a cancer to the presence of certain bacteria (42). To date, CagA from *Helicobacter pylori* has remained the only bacterial carcinogen (43). However, some bacteria are now getting increasing attention, because of the expression of pathogenicity factors with oncogenic potential. For PMT, direct evidence of a link between cancer and *Pasteurella* infections is still elusive despite its manyfold mitogenic actions. While some toxins like the cytolethal distending toxins can directly interact with DNA and cause genomic instability (42), PMT and other toxins like cytotoxic necrotizing factor (CNF) modulate pathways involved in proliferation, apoptosis and replication as well as tumor invasion or immune evasion (44, 45). For PMT, it can be noted that its ability to activate β-catenin, Pim and Akt kinases and mitogenic pathways like MAP kinase and STAT signaling points to an oncogenic potential (44). The activation of the survival kinases Akt and Pim-1 was found to be important for pro-survival signaling and the inhibition of apoptosis in PMT-treated cells (46, 47). Pim kinases are known proto-oncogenes that are activated by the JAK-STAT pathway and consequently PMT intoxication resulted in anchorage-independent cell growth that depended on Pim-1 expression levels (46). As both, Akt and Pim also have important roles in osteoclastogenesis, it is more likely that *in vivo*, the increased activity of these signaling molecules is channeled into osteoclast formation and enhanced resorptive activity of these cells rather than cellular transformation (48, 49). G protein signaling is strongly connected to the Wnt signaling pathway which has important roles in embryonal development of muscle, bone and the heart but also to carcinogenesis. β-Catenin regulates the Wnt signaling pathway through binding to and modulating the activity of transcription factors of the T cell factor family (TCF) (50). In the absence of an activating signal, β-Catenin is degraded in the cytosol. The level of β-catenin is regulated through post-translational mechanisms such as phosphorylation, ubiquitination and subsequent proteasomal degradation (51). Several publications investigated whether G protein activation initiated by PMT creates a direct or indirect link between PMT and β-catenin (14, 52–54). This had first been studied in adipocytes, where it was found that PMT inhibited adipogenesis by enhancing β-catenin stability (53). Developing adipocytes need to down-regulate β-catenin levels to allow for the upregulation of Notch signaling and a cell fate decision towards adipocytes. PMT treatment however, inhibited Notch and stabilized β-catenin, thus causing an arrest of differentiation in the pre-adipocyte state. In the proposed model, PMT enhanced β-catenin expression due to Gα_12/13_ activation and the interaction of RhoGEF with β-catenin (52, 54), but no direct interaction of the toxin with the Wnt pathway was found. More recently, immunomodulatory functions and a role in immune cell homeostasis of β-catenin have also been discussed (55), but it is unknown if PMT-mediated immune escape mechanisms involve changes in beta-catenin levels.

As the prevalence of *Pasteurella* however is low in humans and only a limited number of strains are toxigenic, the likelihood of *Pasteurella*-supported oncogenesis is not concerning, despite the activation of mitogenic pathways. There are reported cases of *P. multocida* infections in cancer patients, however, due to the immunocompromised status transmission might is more likely especially if living with pets and the infection might not be the cause of cancer (42). There are no specific reports available that provide an epidemiological link between bacterial infections and the occurrence of cancer in pigs. However, pigs are often slaughtered at young age so that the development of a cancer might go unnoticed.

## 2 PMT and the immune system

### 2.1 Infection and immunity

The immune system has developed early during evolution, probably with the occurrence of multi-cellular organisms that needed to protect themselves against invaders such as viruses, bacteria or fungi. This innate immune system is restricted to the recognition of differences between self and a potentially harmful non-self that initiates direct defense mechanisms that aim to quickly eliminate the invader (56). The evolution of adaptive immunity is restricted to higher developed organisms and coincided with the occurrence of jawed vertebrates (gnathostomes) around 400 million years ago (57). Adaptive immunity provides a much more specific response to a pathogen through mechanisms of somatic recombination. It is also able to generate memory of previous encounters with pathogens to make the secondary immune response faster and more precise. During an infection, the host responds to the invader with an arsenal of well-constructed weapons. Bacteria also have specific tools that allow them to evade the immune response and to enter their specific niche for replication. The investigation of bacterial toxins and pathogenicity factors has advanced our understanding of the interaction between host and pathogen on the molecular level and the observed cellular effects can now be arranged into the bigger picture of host immunity. In contrast to pathogen associated molecular patterns (PAMPs) that are an integral part of the bacteria and serve as general cues for host cell sensors in innate immune recognition, pathogenicity factors and toxins are produced at specific time-points in a multi-dimensional and multi-factorial manner to actively modulate the reaction of the host. Because of their specificity, bacterial toxins can be exploited as biotechnological tools and the elucidation of their respective mechanism of action added to our understanding of diseases such as cancer or autoimmunity. The elucidation of target molecules, signaling pathways and host responses has shaped our understanding of cell functioning over the past 30 years (58). The better we understand the set of bacterial weapons, the more efficient we can fight infections, which might become more important at a time point where the efficacy of antibiotic treatment is decreasing. Additionally, the broader accessibility of comparative genomics will help to identify unique genes that correlate with pathogenicity or virulence, i.e., the degree of pathogenicity. In addition, these analyses will help us to better understand mechanisms of horizontal transfer and pathogen (re)emergence (59).

#### 2.1.1 Innate immunity – the first responder of immunity

To protect the body against the multitude of microbial organisms, a defense system with several layers is in place. The first layer consists of simple barriers that shield the body from intrusion. These can be mechanical like the epithelial layer of the skin or the lung, of biological nature like skin commensals or consist of chemicals like lysozyme, stomach acid or defensin molecules (60). In case a microbe manages to overcome this first layer, the resulting breach of the barrier leads to an encounter between the microorganism and host cells that are equipped with receptors that allow the body to distinguish between self and non-self-patterns, the pattern recognition receptors (PRR) (56). Upon PRR stimulation, humoral as well as cellular defense mechanisms of local innate immune cells get activated. This is the acute phase of infection which takes place within the first hours after the encounter with the microbial organism. It involves the reaction of preformed humoral systems such as small antimicrobial peptides like cathelicidins and defensins as well the complement cascade, a proteolytic protein cascade of plasma proteins that marks microbes for clearance and kills them through formation of a membrane attack complex and pore formation (60). The cellular first responders of innate immunity are neutrophil granulocytes that make up 50-75% of circulating leukocytes. They phagocytose microorganisms and delete them through the production of bactericidal substances and reactive oxygen species in the phagolysosome. If this early intervention is insufficient to fight back the microbial invader, the early-induced phase is initiated. Chemotactically active substances such as C5a and C3a that were produced by the proteolytic reaction of the complement cascade, bind to complement receptors on innate immune cells so that more leukocytes are recruited. In addition, an increase in complement proteins in the tissue can be detected. C5a and C3a also trigger degranulation of inflammatory mediators like histamine and cytokines from mast cells (61). Cellular defense mechanisms involve phagocytosis and the activation of PRR-induced signaling cascades that result in the production of pro-inflammatory cytokines to further amplify the inflammatory reaction. If a microbe is taken up by phagocytes like granulocytes, macrophages or dendritic cells, microbes will be inactivated through NAPDH oxidase-produced ROS and digested into smaller pieces in the phagolysosome (62). Ultimately, these vesicles are directed towards the ER where major histocompatibility complex II proteins are localized. Once the endolysosomes containing antigen peptides are directed to the ER, MHC molecules get loaded and transported to the plasma membrane where they present the loaded antigen peptide to T cells. Monocytes, dendritic cells and B cells are professional antigen-presenting cells (APCs), but especially dendritic cells are important to activate adaptive immunity through antigen presentation to T cells. This step initiates the late adaptive phase of an immune response which starts around 4 days after the initial contact and leads to the activation of adaptive immunity (18). Circulating dendritic cells (DCs) that took up pathogen and present antigen on their surface migrate from the inflamed tissue to the lymph node, where they can interact with naïve T cells. If a T cell possesses a T cell receptor (TCR) that can bind the antigen:MHCII complex, a T cell response is triggered. Activated T cells produce the mitogenic cytokine interleukin (IL)-2 and a clonal expansion of the activated cells occurs so that within a few days thousands of T cells with the same receptor specificity are produced. Depending on the origin of processed antigen and the surface proteins on the activated APC, cytokines are produced that further shape the immune response (e.g. interferon (IFN)-γ for a cytotoxic cellular response against intracellular invaders, or IL-4 for an antibody-based humoral response against extracellular invaders). To trigger B cell-mediated antibody production, a B cell receptor (BCR) has to recognize the same microbe that activated the TCR. Once the BCR is endocytosed with its cargo, the antigen can be presented to T cells at a specific site in the lymph node. This will cause the formation of follicles where B cells optimize the antibody against the microbe and eventually produce antibodies. Once antibody producing plasma B cells secrete antibodies, they get transported to the site of infection *via* the blood stream (**Figure 2**). B and T cells also differentiate into memory cells that will help to identify an invading microorganism even years after the first infection happened.

#### 2.1.2 Bacterial pathogenicity factors – a short introduction

In many cases, the immune system is able to effectively clear the infection so that the intrusion of a microbe might even happen unnoticed. However, bacteria have a fascinating toolbox of pathogenicity factors that allows them to effectively hide from detection through the immune system or to manipulate host cells into an anti-inflammatory state to establish a niche for their survival and replication. From the perspective of the microbial invader, the process of infection starts by finding a mechanism that allows the microbe to enter the body unnoticed (63). Specific surface structures like capsules shield it from the recognition by PRRs, enlarge the microbe so that phagocytosis is prevented and cause electrostatic repulsion (63). Additionally, the carbohydrates of the capsule make it more difficult for immune cells to adhere and establish a contact that allows to attack the invader. Thus, capsule bearing bacteria are more virulent than non-capsule strains of the same species, which is also found for *Pasteurella multocida* (6). Lipopolysaccharide (LPS) of Gram-negative bacteria makes cells less accessible for complement-mediated lysis and variation in the LPS formation (O-antigen) decreases recognition by toll like receptors (TLRs) which allows the bacteria to go unnoticed by TLR4 recognition (64).

To establish a niche for replication, effector proteins are produced that are injected into the host cell using a specific secretion system. These pathogenicity factors not only promote bacterial colonization and survival, but are often able to damage the host. The production of pathogenicity factors is organized in space and time, which means their production happens at a specific time-point of invasion and only at specific localizations. Pathogenicity factors often target important intracellular signaling cascades like NF-kB and MAPK signaling to limit the production of defensins and cytokines (65). The inhibition of the secretory pathway of cells is another powerful tool to limit the immune response, as humorally acting substances need to be transported intracellularly and secreted to the outside to be effective (63). Exotoxins are a specific group of pathogenicity factors that are secreted by the bacteria but do not require a secretion system for cellular uptake. Often, these toxins originate from bacteriophages (66). As the acquisition of virulence genes is a central factor that determines how effective a microbe is in establishing a niche in its host, there are a number of ways that allow bacteria to exchange DNA horizontally. Horizontal gene transfer can occur *via* transformation, i.e., the uptake of naked DNA, viral transduction by bacteriophages, or conjugation for cell mating through specialized pili. These horizontally acquired genes are often unique to pathogens and localized on plasmids, prophages or transposons which can be identified by boundary sequences using comparative genomics (67). Such acquired genes or groups of acquired genes have differing GC content, strand bias or codon bias that differs from the rest of the genome or the immediate surroundings. Transfer of such a virulence gene to new cells can even occur across species, thus conferring virulence to formerly benign strains.

There are three types of bacterial exotoxins, that can be grouped into pore-forming toxins (type I), superantigens (type II) and AB toxins (type III). Pore-forming toxin can be enzymatically active proteins (e.g. lipases) or channel forming toxins like cholesterol-dependent cytolysins (CDC) and the RTX toxins. Often, the function of these toxins is not limited to the simple killing of a cell. CDCs can affect innate immune signaling dependent on cholesterol-rich microdomains resulting in the downregulation of proteins like TLR4 or CD86 that have important roles in pattern recognition and antigen presentation (68). The repeat in toxin (RTX) family is produced by many gram-negative bacteria and hemolysin A (HlyA) of *E. coli* is a prototypical example. RTX toxins are characterized by nine amino acid glycine-aspartate-rich stretches that are tandemly repeated which helps them to assemble into a calcium-loaded beta-roll structure (69). After modification by acyl transferases, RTX proteins insert into the cell membrane which results in cell damage. RTX-mediated attack of phagocytes then triggers the release of pro-inflammatory factors resulting in further tissue damage, inflammasome-mediated pyroptosis and the release of danger associated molecular patterns (DAMPs). The toxin breaks down epithelial barriers to establish a niche for the bacteria to persist, but it was observed that RTX toxins can also subvert signaling cascades of the host cell, especially when the pore is used to deliver further pathogenicity factors (69, 70). Superantigens are type II exotoxin that do not enter host cells. Instead, they unspecifically link MHC molecules to T cell receptors in an antigen-independent way, causing a massive activation of T cells and the subsequent release of IFN-γ. The resulting hyperactivation of macrophages triggers the production of tumor necrosis factor (TNF)-α which can lead to hypotension and shock in the patient (71). However, not much is known on the direct effect of the superantigen binding on the dendritic cell. It was suggested that endocytosed superantigens do not trigger DC maturation and antigen processing but that the proteins remain active and get recycled to the cell surface again (72). Others found that although superantigen binding caused the release of pro-inflammatory cytokines by T cells, a tolerizing program through upregulation of IL-10, PD-L1 and IDO was initiated by the DCs (73).

AB exotoxins constitute the third class of exotoxins and enter host cells through receptor-mediated endocytosis. The endosome containing the endocytosed receptor-toxin complex fuses with the lysosome and acidification of the compartment induces a conformational change of the toxin so that it can get translocated into the cytosol (74). Exotoxins therefore only attack cells with a corresponding receptor. In contrast to pathogenicity factors, which require the presence of living bacteria, bacterial toxins can exert their function once released from the pathogen and often the toxin is sufficient to cause disease. Many bacterial toxins target molecules of the host that are central for the cell to survive, replicate or exert its function, for example actin, Rho GTPases or heterotrimeric G proteins (74–76). Typical modifications are ADP ribosylation (74), glycosylation (77), but other functional modifications that can change the activation status of the target molecule, like deamidation (78) are also described. Initially, the focus of toxin research was on the cellular microbiology of the immediate molecular effects of a toxin on a host cell. As ultimately toxins are employed by the bacteria to create a niche for survival, to kill the cell, or to subvert the immune response, the function of bacterial toxins must also be seen in the context of host immunity. As most if not all bacterial toxins affect the cytoskeleton, typical toxin-mediated effects include inhibition of phagocytosis and destruction of the pathogen as well as an impact on the subsequent antigen presentation *via* MHCII molecules and the failure to induce an adequate adaptive immune response (79). However, a detailed and up to date review of type III toxin-mediated effects on leukocytes is missing and a comprehensive review on the effects of major toxins by Cubillos et al. shows that many of the cited papers are more than 30 years old (79). The last few decades have been a very successful time for the field of immunology with the discovery of new Th subsets like Th17 cells (80), regulatory T cells (81), or the discovery of Toll like receptors (82) and the inflammasome platform (83). Although toxins often target specific cells through the use of specific host cell receptors for endocytosis and may not specifically target immune cells, the resulting toxin-induced damage will eventually trigger leukocyte activation. A recent example is the activation of the Pyrin inflammasome through toxin-induced glucosylation of Rho GTPases (84). Such discoveries have broadened our horizon in the understanding of the interplay between toxin activities and immune reactions during the last decade and help to connect and transfer the data obtained in cell line model systems to *in vivo* model systems. Therefore, there is a need for an updated understanding of the impact of toxins on the host immune response.

### 2.2 *Pasteurella multocida* toxin as a modulator of immune reactions

PMT is best known for its differentiating effect on macrophages and monocytes into bone resorbing osteoclasts. However, it was found that PMT also directly influences the signaling and the immune reactions of innate immune cells like macrophages and dendritic cells. Interestingly, despite their functional similarities, there was a clear difference between the PMT-mediated impact on macrophages and DCs with respect to the production of cytokines and osteoclastogenesis. While PMT induced persistent production of pro-inflammatory cytokines in macrophages which differentiated readily with PMT even in the absence of the differentiation factor receptor of activated NF-κB (RANKL) (35, 85), PMT inhibited RANKL-mediated osteoclast differentiation in bone marrow-derived DCs (86). In DCs, only TNF-α was produced, which has a weak osteoclastogenic activity. Instead, a strong induction of CD80 and CD86 expression upon PMT treatment was observed, which was absent in macrophages (86). Despite the fact that macrophages seemed to prefer differentiation over activation, also PMT-treated macrophages are able to trigger an immune response. Most importantly, a novel, non-canonical mechanism of IL-1β production that takes place without the induction of pyroptosis was found. Here, PMT-induced IL-1β production occurs through pro-IL-1β cleavage through the serine protease Granzyme A (87), instead of canonical pro-IL-1β cleavage through the inflammasome-dependent activation of the cysteine protease caspase-1. Interestingly, this was not followed by cell death neither through apoptosis nor pyroptosis and it was speculated that PMT-induced anti-apoptotic pathways might be the reason for this unusual observation (87). It was later described that pyroptosis is actually not caused by IL-1β directly, but is the result of Gasdermin D-mediated pore formation which occurs through caspase-1 proteolytic cleavage of Gasdermin D (88). Since caspase-1 is not activated by PMT, this is a likely explanation for the absence of cell death after PMT treatment and an exciting example how bacteria subvert the immune response.

An important aspect of innate immunity is the recognition of microbial pathogens through pattern recognition receptors, such as Toll like receptors (TLRs). Using recombinant PMT, it was found that in human monocytes, PMT was not able to induce the expression of pro-inflammatory cytokines such as TNF-α or IL-12 and that the production of IL-6 and IL-10 was much lower than for LPS (89). The suppression IL-12 production from human monocytes subsequently also impaired the ability of these cells to activate T cell proliferation (89). Inhibitor experiments showed that this effect was mediated *via* PMT-induced Gα_i_ signaling. In bone marrow-derived dendritic cells (BMDC) from mice the production of cytokines was also reduced, however here, the treatment of BMDCs with PMT was found to enhance the production of IL-12, which in addition to the ongoing maturation process of the DCs might be another reason for the observed suppression of osteoclastogenesis (86).

Stimulation of bone marrow cells with PMT in the absence of any other growth factor preferentially yielded B cells and macrophages (90). The osteoclasts obtained at later stages from these cell cultures arose from macrophages, but not haematopoietic progenitor cells, as CD117^+^ stem cells themselves were not reactive to PMT. Cocultivation of purified B cells and CD11b^+^ macrophages strongly enhanced osteoclastogenesis, presumably through the expression of the cytokine RANKL on the surface of B cells. In addition to its interaction with B cells, PMT also has a direct effect on T cell differentiation (91). Stimulation of activated, naïve T cells resulted in the activation of both, Foxp3 and RORγT, the transcription factors that trigger the differentiation of Tregs and Th17 cells. This is an unexpected finding as these two T cell subsets have opposing functions. While Th17 cells are connected with the eradication of extracellular bacteria, persistent infections and autoimmunity, regulatory T cells suppress the immune response. Functionally, these cells seemed to resemble Th17 cells as the cells produced high amounts of IL-17 like normal Th17 cells. In contrast to Tregs, PMT-treated T cells were not able to produce IL-10 and thus did not have an anti-inflammatory phenotype. The Th17 subset is in addition strongly connected to a bone phenotype as it expresses RANKL (92). Whether the described Foxp3/RORγT T cells are fully functional with respect to the ability to react against extracellular bacteria *in vivo* has not been investigated.

## 3 PMT-mediated effects on specific organs

The best described effect of PMT is its drastic impact on bone homeostasis which results in a twisting and shortening of the snout due to the loss of bone material. The presence of the toxin is sufficient to cause this pathology and does not require the presence of bacteria (93, 94). Not much is known about other target sites of the toxin in the host. Recently, it was investigated which organs PMT targets *in vivo* by making use of an antibody that detects the deamidated Gα_q_ (14, 95). Tissues from spleen, lungs, thymus, gonads, heat, bone, liver colon, bladder, skin were investigated with a focus on pathways that play a role for cell proliferation. Intraperitoneal injections of PMT for three weeks caused weight loss in the mice, however, the animals still appeared healthy and a post-mortem analysis did not reveal an overt phenotype at the macroscopic level. Many organs showed deamidation of Gαq but the highest levels were found in the spleen, lungs, thymus and gonads. As already seen in tissue culture experiments, systemic delivery of PMT went along with increased mitotic activity in the targeted tissue. Active β-catenin for example was found in the endometrium, in luminal epithelial cells of the red pulp of the spleen and the medulla of the thymus, suggesting that β-catenin might indeed have immune-modulatory functions in this context (55).

### 3.1 Heart

G proteins are highly important for the activation of adrenoceptors in the heart muscle under physiological and pathological conditions (96). Cardiac hypertrophy is a common consequence of myocardial infarction, ultimately leading to chronic heart failure. In an attempt to restore cardiac output, the GPCR ligands catecholamines are secreted. Although these adaptive mechanisms of α and β-adrenoceptor activation initially preserve cardiac output, with time excessive compensatory remodeling results in tissue scarring and cardiomyocyte hypertrophy that damages the organ and promotes heart failure.

Moderate overexpression of Gα_q_ had been shown to cause cardiac hypertrophy while a strong increase in Gα_q_ levels resulted in cardiomyocyte apoptosis (97). PMT was used as a tool to study the effect of G protein signaling in a model system that is independent of over-expression. In an early study, it was found that PMT treatment of neonatal rat myocytes and cardiac fibroblasts constitutively activated Gα_q_ signaling that resulted in cardiomyocyte hypertrophy and enhanced susceptibility to apoptosis due to the inhibition of Akt signaling (98). To investigate the mechanistic details further, intraperitoneal injection of PMT was used to study the systemic effects of PMT in mice. Using a deamidation-specific antibody, it was found that PMT causes Gα_q_ deamidation of both, cardiac fibroblasts and cardiomyocytes (99). When the mice were co-treated with PMT and the α-adrenoceptor agonist phenylephrine, an increase in ventricle weight and fibrosis could be observed as early as eight days after injection. In cardiomyocytes, pathways with a known role in cardiac hypertrophy, like MAP kinase signaling and Rac1 and RhoA activation, were found to be triggered by PMT (99). As previously described for the fibroblast cell line 3T3, where the activation of mTOR as a consequence of PMT treatment was investigated (100), connective tissue growth factor (CTGF) production was also increased in cardiac fibroblasts (99). Interestingly, CTGF is a growth factor used as a biomarker that is induced after cardiac injury and plays a role in cardiac fibrosis (101).

Especially in the context of myocardial infarction PMT might also have beneficial functions if applied locally, due to its anti-apoptotic function. In contrast to PMT-mediated systemic effects during an infection, here, Gα_q_ signaling would only be activated for the respective lifetime of the deamidated G protein. Due to the activation of the JAK-STAT pathway and the subsequent induction of the survival kinase Pim-1, cardioprotective pathways might be triggered. Indeed, Pim-1 gene therapy has been assessed for its applicability as a means to rejuvenate cardiac progenitor cells (102). Further studies concerning the PMT-mediated effects in cardiomyocytes would be helpful to investigate if PMT could act as a stimulus that shifts cellular signaling towards survival and regenerative mechanisms which could limit progressive remodeling and the transition into heart failure.

### 3.2 Brain

Limited information is available regarding the effect of PMT on neuronal cells. For an investigation of the binding mechanism of PMT to membrane lipids, the mouse neuroblastoma/rat glioma hybrid cell line NG108-15 was used (24). It was found that the toxin, if it crossed the blood-brain barrier, would in principle be able to interact with neuronal cells (24). Because heterotrimeric G proteins are important players in the signaling processes of neurons, Surguy et al. investigated the effect of PMT in a rat model (103). Using rat superior cervical ganglion neurons (SGC) and the neuronal cell line NG108-15, the authors showed that PMT did not act on the voltage gated channels Kv7 and Cav2 directly. Instead, PMT enhanced the muscarinic receptor-mediated inhibition of the M current which involves Gα_q_ signaling and PIP_2_ release. This seemed to be a stimulus-dependent effect, as the effect of Bradykinin and Angiotensin which also block the M-current was not enhanced by PMT. However, this was only seen in the presence of GPCR stimulation with a pharmacological agonist but not for PMT stimulation alone. The effect of PMT on calcium channels was investigated in transduced NG108-15 cells and again PMT was able to enhance the effect on calcium mobilization when the purinoreceptor P2Y_2_ was stimulated with UTP, but not by the toxin itself.

These cell culture data suggest that there is the possibility that the toxin, if located in the brain, could trigger hyperexcitability and seizures (103). Indeed, for pigs that had been naturally infected with the non-toxigenic B:2 strain, neuro-invasion of Pasteurella was seen (104). Whether serotype specific differences exist is currently unknown. In humans, were *Pasteurella* infections are rare, meningitis and neurological complications have also been reported. Such infections with complications mainly occur in pediatric or elderly patients but rarely in adult immune competent humans. The source of infection can in most cases be traced to close interactions with household animals such as cats and dogs and should therefore mostly be associated with non-toxigenic strains, but accurate data are missing.

### 3.3 Bone

In addition to its function as a structural organ that is required for mobility, hematopoiesis and calcium storage, bone is also an endocrine organ that impacts metabolic processes through the production of the peptide hormone osteocalcin (105). Bone material is generated and maintained through the interaction of two types of bone cells called osteoclasts and osteoblasts. Osteoblasts develop from the mesenchymal lineage and build bone, while osteoclasts are haematopoietic cells that develop from the monocyte/macrophage lineage and can therefore be regarded as immune cells (106). As osteoclasts differentiate from macrophages and pro-inflammatory cytokines like IL-6, TNF-α and IL-1β also support osteoclastogenesis, there is a strong mutual regulation of these cells (107, 108). The importance of osteoclasts as immune cells is less well understood (109), but examples can be found in the literature that show that osteoclasts maintain immune cell activity. They can for example serve as APCs and interact with T cells (110) but osteoclast progenitors can also be turned into myeloid-derived suppressor cells that actually reduce a T cell response (111).

#### 3.3.1 Osteoblasts

**Figure 3:**
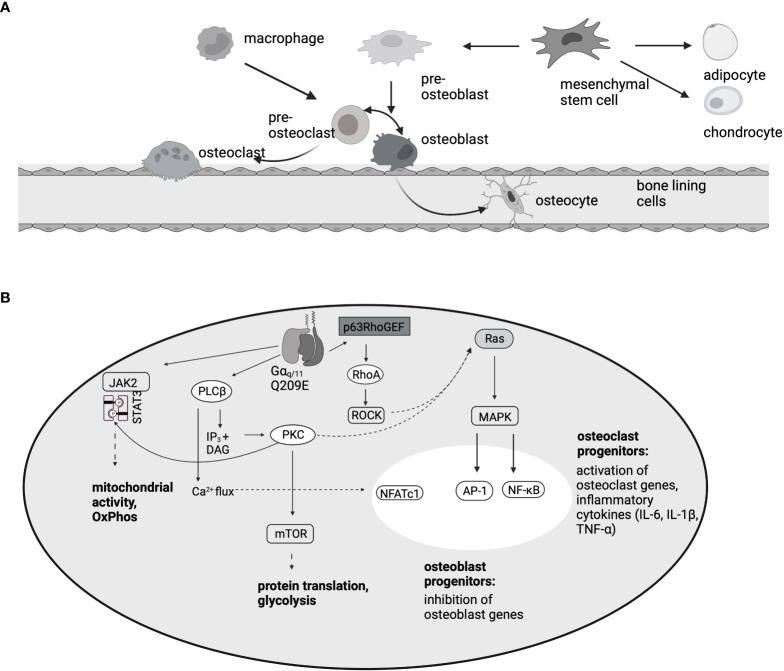
PMT-mediated effects in the bone. **(A)** Osteoblasts, like chondrocytes and adipocytes, develop from mesenchymal stem cells. Osteoclasts are haematopoietic cells that develop from macrophage/monocyte precursors. This is mediated by the two cytokines M-CSF and RANKL. Because RANKL is expressed on osteoblasts, there is a direct interaction between the two cell types. **(B)** Summary of the pathways involved in osteoclast differentiation and inhibition of osteoblastogenesis, according to data published by (35, 85, 112, 113). In osteoclast progenitors, activation of the transcription factors NFATc1, AP-1 and NF-κB results in osteoclast gene transcription, however, in osteoblast progenitors, MAPK activation inhibits the induction of osteoblast differentiation genes.

Osteoblasts are derived from mesenchymal stem cells and act as bone building cells (106). Because bone formation and bone degradation are strongly connected, osteoblasts express RANKL on the surface, which stimulates osteoclast differentiation (106) (**Figure 3**). In ST-2 cells, PMT specifically blocks differentiation of mesenchymal osteoblast progenitors at concentrations as low as 10 pM, but does not interfere with the differentiation of other mesenchymal-derived lineages like adipocytes (112). Mechanistically it was shown that Gαq triggers p63RhoGEF-mdiated activation of RhoA and MAP kinase signaling (112). RhoA seems to be the central molecule of the cascade, as intoxication of the cells with the exotoxin CNFy from *Yersinia pseudotuberculosis* that directly targets and constitutively modifies RhoA also blocked osteoclastogenesis. Similarly, PMT is able to inhibit osteocytes. Osteocytes are osteoblasts that were integrated into the bone material during the process of bone regeneration. In MLO-Y4 osteocytes, PMT-mediated G protein activation caused stress fiber formation and inhibited the RhoC-mediated dendrite formation that is typical for osteocytes (114). Additionally, RANKL, the cytokine that is crucial for the stimulation of osteoclast differentiation, as well as osteoclastogenic inflammatory factors such as TNF-α, were released from the cells. The use of a co-culture model indeed showed that PMT enhanced the interaction between osteocytes and osteoclasts, swinging the balance in favor of osteoclastogenesis and bone loss (114).

Fibrodysplasia ossificans progressiva (FOP) is a rare human disease with only around 700 patients worldwide, characterized by the progressive ossification of skeletal muscle, fascia, ligaments, tendons, and soft tissue into bone and accordingly is highly disabling (115). The pathology is caused by a point mutation within ACVRI (Activin A receptor, type I), a bone morphogenic protein (BMP) type 1 receptor of the TGF-β receptor superfamily (116). This point mutation renders the receptor reactive to the antagonistic ligand Activin A and thus causes enhanced bone formation. In a study by Ebner et al., C2C12 myoblasts transduced with the mutant receptor were used as a model for the disease and the ability of PMT to influence and inhibit the trans-differentiation pathway was investigated (117). Incubation of C2C12 cells with BMPs had previously been shown to cause trans-differentiation into osteoblasts (118). Due to the inhibitory effect of PMT on osteoblastogenesis, PMT-mediated Gα_q_ activation or activation of Gα_q_ *via* adrenoreceptors blocked the Activin A-induced stimulation of mutant ACVRI and the subsequent release of alkaline phosphatase (ALP), an early osteoblast differentiation marker, highlighting the translational potential of the toxin.

#### 3.3.2 Osteoclasts

Osteoclasts are haematopoietic, monocyte/macrophage-derived cells that differentiate in the bone through the action of two cytokines, macrophage colony stimulating factor (M-CSF) and RANKL, and have the unique ability to resorb bone material. During inflammatory conditions, especially when chronic, bone loss can be observed due to increased osteoclast formation and activity. Osteoclasts can also develop from DC as they share a common progenitor, but it needs to be further investigated whether there are functional differences between these cells (119, 120). Under normal bone homeostasis, DC do not appear to contribute to bone remodeling as no skeletal abnormalities were observed in a DC-deficient mouse model (121). However, in inflammatory bone diseases DC can be found at the site of inflammation (121, 122).

PMT induces OC differentiation in RAW264.7 macrophages as well as in CD14^+^ monocytes or bone marrow-derived macrophages (35, 85, 123). Mechanistically, mTOR activation is a central process for osteoclastogenesis triggered by RANKL treatment (124). Similarly, rapamycin treatment strongly inhibited the formation of PMT-induced TRAP-positive osteoclasts, but did not affect the production of proinflammatory cytokines or proliferation (123) (**Figure 3**). The central transcription factor for RANKL-mediated osteoclast differentiation is NFATc1 (125). PMT is able to cause NFATc1 activation *via* Gα_q_ signaling and Gα_q_ inhibition abrogated NFATc1 transcriptional activity and osteoclast formation (35). Other transcription factors that support osteoclast formation like AP-1 and NF-kB were also found to be upregulated by PMT (35, 123). Following work by Chakraborty et al. showed that PMT creates a microenvironment where NFATc1 activation is accompanied by the production of pro-inflammatory cytokines such as IL-1β, IL-6 and TNF-α and that the combination of the two queues is needed for osteoclast formation in the absence of RANKL (85). Indeed, inhibition of TNF-α or IL-6 strongly suppressed the ability of PMT to trigger osteoclastogenesis, despite the constitutive activation of NFATc1. Through a differential 2D gel electrophoresis (DIGE) approach it was recently shown that PMT treatment causes the upregulation of proteins involved in glycolysis and metabolic pathways (113). This seems to be caused by deamidation of Gα_q_, as overexpression of Gα_q_ mimics this hypermetabolic phenotype and results in increased resorptive activity. These data suggest that Gα_q_ might have a function in bone remodeling, at least under pathological conditions and that it might be worthwhile to investigate the effect of Gα_q_ in more detail. Interestingly, rheumatoid arthritis patients also showed an increase in *Gnaq* expression (113), suggesting that this finding is of pathophysiological relevance.

## 4 What have we learned – what are the perspectives

Toxins have been recognized as powerful tools to study host cell processes and to possess translational potential that can be employed to modulate cell signaling events. Traditionally, toxins and their toxoid forms have been used as vaccines as they are often the central players in the disease and confer effective protection from the disease. Since the targets and mechanism of bacterial toxins have by now been characterized in detail, protein domains of interest can be further engineered to fit specific purposes. If the mechanism of toxin uptake can be directed to a specific cell, the catalytic activity needs to address a wanted function in the target cell (126). Ontak is an example of an engineered IL-2-diptheria toxin that targets IL-2 receptors on cancer cells into which diphtheria toxin is then delivered. Due to the inhibition of cellular translation by the toxin, the cancer cell quickly dies (126). For PMT, a minimal region was identified that is sufficient to deliver diphtheria toxin inside a target cell (127). Similar results were published by another group that showed that the N terminus of PMT can be used to successfully cargo GFP into the cytosol in the presence of the C1 and C2 domain of the natural cargo (128). While the use of PMT as a delivery tool is unlikely because of the abundant uptake by most types of host cells, these experiments are important to optimize the combinations of cargo and the shuttle for optimal delivery of the cargo inside the cell that guarantees proper folding and functional activity in general.

PMT has rather unique cellular functions due to its mitogenic properties. This has raised concerns whether the toxin might be able to act as a bacterial carcinogen (129) and also limits its therapeutical use to scenarios where the prevention of cell death is of interest. This includes pathologies such as stroke or myocardial infarction where the PMT-induced upregulation of the survival kinases Pim and Akt might be beneficial. Indeed, Pim kinases have been discussed as suitable gene therapy targets to avoid tissue scarring and prevent chronic heart failure in patients after myocardial infarction (102). Furthermore, PMT was suggested for the treatment of Fibrodysplasia ossificans progressive (117), as it counteracts excessive bone formation. More importantly, PMT has added a wealth of information on our understanding of G protein signaling and the recent finding that Gα_q_ is an important regulator of cell metabolism and might be involved in autoimmune diseases like rheumatoid arthritis (113) shows that research on bacterial toxins has the potential to make important revelations in the field of microbial immunology and human diseases.

## Author contributions

KK searched the literature, designed the review and wrote the manuscript.
